# Participatory Research for Preventing Pesticide-Related DSH and Suicide in Sundarban, India: A Brief Report

**DOI:** 10.1155/2013/427417

**Published:** 2013-04-23

**Authors:** Arabinda N. Chowdhury, Sohini Banerjee, Arabinda Brahma, Mrinal K. Biswas

**Affiliations:** ^1^Stuart Road Resource Centre, Northamptonshire Healthcare NHS Foundation Trust, Corby, Northants NN17 1RJ, UK; ^2^Institute of Psychiatry, 7 D. L. Khan Road, Kolkata 700025, India

## Abstract

Deliberate self-harm (DSH) is a major public health problem in the Sundarban region, India. This study is aimed to develop a DSH-suicide prevention programme based on the principles of community-based participatory research (CBPR). Perception and opinion of community about the problem of pesticide-related DSH and suicide were elicited in a series of facilitated focus group discussions in Namkhana block of Sundarban region. Based on their suggestion, a broad preventive programme was launched involving the development of information, education, and communication (IEC) and training modules and training of the stakeholders of the block. Most of the members of each target group found that the IEC materials were *culture fair* (message is acceptable, understandable, and meaningful in the local context) and very useful. Analysis of Dwariknagar BPHC, DSH admission data showed a definite reduction of DSH incidents after this CBPR approach to prevention was initiated. Similar model of DSH prevention in the other blocks of Sundarban region or in agricultural community may help to reduce the enormous mortality and morbidity from pesticide-related DSH and suicide.

## 1. Introduction

Deliberate self-harm (DSH), both fatal and nonfatal, is one of the most challenging public health issues in the Sundarban region, West Bengal [1, 2]. It is reported that one-year incidence rates from population surveys of nonfatal DSH ranged from 700 to 1,100 per 100,000 people, and life-time prevalence rate ranged from 720 to 5,930 per 100,000 persons [3]. Nonfatal DSH (or nonsuicidal self-injury) is 10 times more common than suicide [4]. In India, about 100,000 persons commit suicide every year, which is approximately 10% suicides in the world [5]. Suicide is among the top ten causes of death in India and among the top three causes of death among those between 16 and 35 years [6]. 

Indian research on DSH [7–11] has focussed on the positive association of various sociocultural and environmental factors with suicidal behavior, thereby highlighting the importance of community-based psychosocial intervention. Suicidal deaths are preventable if sufficient knowledge and understanding of this maladaptive behaviour can lead to timely intervention. Recent suicide prevention programmes address this intervention aspect as one of the top priority public health agenda. The present study is an attempt at understanding DSH behaviour, both fatal and nonfatal, and its preventive approach from the community perspective at a primary care setting and thereby devising a preventive methodology based on community-based participatory research in the remote Sundarban Delta region.

Sundarban is the southern coastal region of the Indian state of West Bengal and is the largest delta of the world (combining with Bangladesh). The West Bengal portion comprises 19 community developmental blocks—13 in the district of South 24 Parganas and the rest in North 24 Parganas. Each block has one main clinical facility—a block primary health centre (BPHC). There are 54 islands spread over an area of 1630 square miles, intersected by numerous canals and tidal creeks. Human habitation is protected by man-made earthen embankment of over 3500 km. Majority (88.5%) of the inhabitants are dependent on agriculture and fishing (about 15%). The ecological character of this region is largely responsible for its remoteness, backwardness (human development index—0.55 in contrast to 0.62 of the district), and varieties of psychosocial community stresses (climatic insults and natural calamities like storm, cyclone, inundation of saline water into the agricultural field, crop failure, poverty, domestic violence related with dowry demand, alcoholism and gender discrimination, etc.) [12]. Both the literacy rate and per capita income are much lower than the state average.

**Figure 1:**
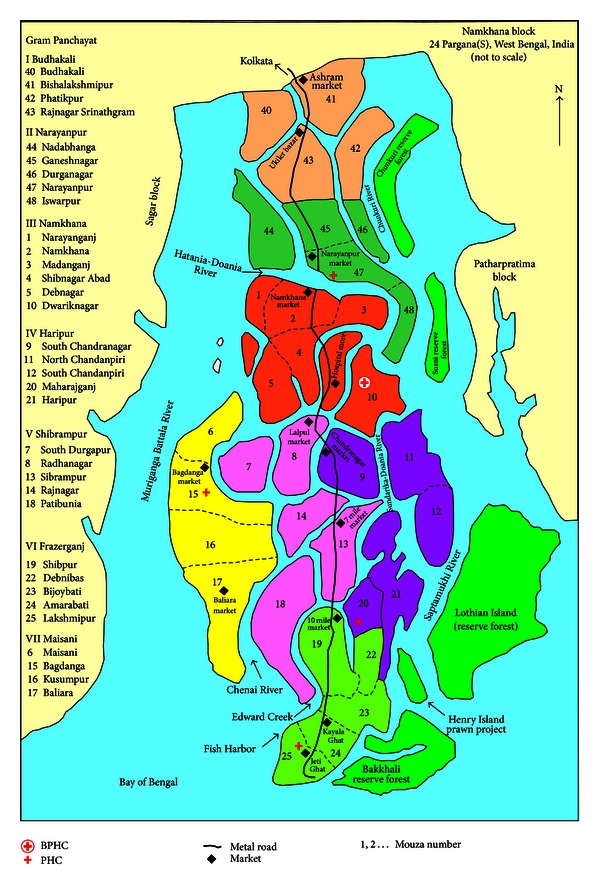
Namkhana block (34 villages under 7 GPs), Sundarban.

In recent years, pesticide-related deliberate self-harm, both fatal (suicide) and nonfatal, was recognised as an emerging public health problem in the Sundarban [13]. We carried out a 2-yr retrospective (2000-01) and 4-yr prospective study (2002–05) of DSH cases (both fatal and nonfatal) admitted to Dwariknagar BPHC of Namkhana block (Figure 1) of Sundarban under South 24 Parganas district, as a part of ongoing community mental health service programme in the region [14]. The objective of the present paper was to follow the community-based participatory research (CBPR) and thus to assess the perception of community (e.g., farmers and Gram Panchayat members (GPMs)) of the Namkhana block about the causes and potential solution to prevent the enormous load of morbidity and mortality from DSH suicide in the region. The insight gained by studying community's perception and opinion about pesticide-related DSH suicide was utilised to develop community action agenda including training modules and IEC materials on DSH-suicide prevention. 

## 2. Materials and Methods

### 2.1. Ethical Approval

The study protocol was approved by the Ethical Committee of Department of Health and Family Welfare, Government of West Bengal and Panchayat Samity, Namkhana Block, South 24 Parganas.

### 2.2. Study Area

Namkhana is the most southbound island block of eastern Sundarban region at the Bay of Bengal with a population of 160627 (2001 census) and 7 Gram Panchayat and one Panchayat Samity—members of all are democratically elected for local self-government units. The main livelihood measures are 80% farmers, 15% fishing, and 5% others (service, school teachers, etc.). There is one block primary health centre at Dwariknagar and 4 primary health centres (PHCs) (from North to South Narayanpur, Maisani, Haripur, and Frazerganj) in Namkhana. Different categories of health staff, both governmental and nongovernmental provide health care to the community. 

### 2.3. Facilitated Focus Group Discussions

A series of FGDs was conducted with farmers Panchayat members, school teachers, students, health staff, integrated child development scheme (ICDS) workers, multipurpose health workers (MPHWs), local health care providers (HCPs) (there is an intricate network of health care providers, who are non-registered practitioners, locally known as “Quack” and virtually they are the first line of contact for the vast majority of Sundarban people [15]), members of NGOs and Mahila Samity (women activist groups), and pesticide shop owners of Namkhana block. The data relating to only farmers and Panchayat members are presented here. 140 farmers (20 from each GP-selected by the Gram Panchayat Pradhan), 124 Gram Panchayat members (of all the 7 GPS), and 23 members of Panchayat Samity (three-tire Panchayat system: blocks are the definite administrative area under a district. Each block has many Gram Panchayats (covering 4–7 villages or mouza each) under one Panchayat Samity. At the district level there is one Zila Parishad. Members of all these are democratically elected) were interviewed in a facilitated focus group discussion, planned in a predetermined date and place. The farmers were selected by the respective GP Pradhan (head), and all the Panchayat members are elected members of the Panchayat body. The themes of the FGDs were “problem of pesticide-related DSH in the block and how to prevent it.” The FGD was facilitated with a short questionnaire “opinion about pesticide-related DSH event in your locality” (6 items) at the beginning of the session, which was helpful to offer the direction of the FGD to the participants. Each FGD was spanned from 1.5 to 2 hours. The FGD responses were recorded with their permission and transcribed afterwards. The responses were analysed as per the main issues and presented in the result section.

### 2.4. Development of IEC and Training Materials

Based on their suggestions, a handful of IEC materials and training module addressing the DSH prevention were devised in local vernacular (Bengali). For each of the materials, the drawings, content, format, and language were consulted with community groups in minute detail, so that they would be culture conducive and transmit health message in an acceptable way to the target groups. In fact, most of the contents were the verbatim of local people on these issues. 

Following is the list of IEC materials devised for this community-based DSH prevention in Namkhana:

- five booklets: on mental health; DSH prevention; psychosocial intervention; violence towards women; safe use of pesticides and common unsafe use of pesticides,
- five WHO suicide prevention booklets translated into Bengali (approved by WHO): preventing Suicide—for general physicians; for primary health care worker; for teachers and school staff; for media professionals and for survivor group,
- nine posters: on prevention of DSH suicide; on mental health; prevention of alcoholism-dowry torture; accidental pesticide poisoning; pesticide cycle; on violence towards women; emergency steps for treatment of pesticide poisoning,
- three folders: on mental health; pesticide safe use; pesticide poisoning and DSH,
- five leaflets: on DSH-suicide prevention; alcoholism; domestic violence; mental health; and safe use of pesticides (good pesticide practice—GPP),
- eight stickers: on DSH-suicide prevention; dowry torture; violence towards women; alcoholism; health hazards of pesticides; safe use of pesticides; safe storage of pesticides; mental health,
- three wall calendars and 3 pocket calendars: with DSH-suicide prevention message for distribution to members of Panchayat; NGO; Mahila Samity; teachers; schools and HCPs,
- one wall clock: with engraved message to prevent pesticide-related DSH suicide, distributed to all the 8 Panchayat offices, 12 secondary schools and 9 NGO, and 6 social clubs of the block.

A feedback questionnaire (yes/no response) was used for each IEC material from each target group, randomly selected, to assess the culture fairness (design, drawings, language, content and message, and overall display) and usefulness (in skill and knowledge development and in DSH prevention community work) of the IEC.

## 3. Results 

### 3.1. Farmers' Responses and Opinion

A total of 140 farmers attended the 11 facilitated FGDs. Pesticide-related suicidal behaviour was acknowledged as an important health problem by 76.7% farmers. 53.3% farmers store pesticide at home and among them 31.3% kept it open. 73.3% farmers purchase pesticides a week before the use. 36.7% had no knowledge of its ill effects on crops and 53.3% of its deleterious effects on the environment. 65.2% farmers said that the public education and 30.4% said that the safe storing of pesticides should be the main preventive approach against DSH-suicide in the island. 92% acknowledged the need for training on pesticide safety and good pesticide practices.

### 3.2. Gram Panchayat Member's Responses and Opinion

**Figure 2:**
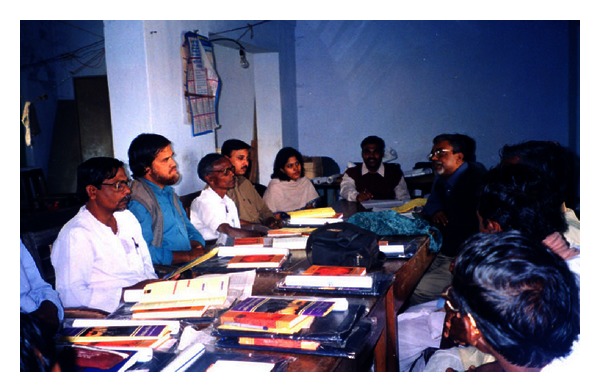
FGD at Maisani Gram Panchayat, Namkhana block.

**Figure 3:**
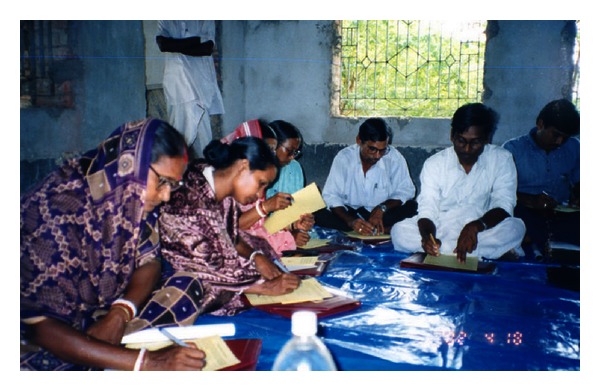
FGD at Haripur Gram Panchayat, Namkhana block.

7 FGDs were conducted at respective Gram Panchayat offices (Figures 2 and 3). 95% GPM knew someone with pesticide-related DSH or suicide in their respective villages. 86.3% endorsed it as a health problem. 88.7% GPM believed that GP has a definite role in DSH-suicide prevention by developing farmers training on safe pesticide use, active involvement of local agriculture department, reducing domestic violence, and imposing strict vigilance on pesticide sale. 80% opined that GP should be empowered to regulate pesticide sale in their jurisdictions. 97% GPM felt the need for mental health training including DSH prevention to its members as also to different sections of the community.

### 3.3. Panchayat Samity Members' Responses and Opinion

One FGD was conducted at Samity office. 98% of the members said that they had no discussion on mental health ever in their Panchayat Samity, and all of them (100%) opined that Panchayat needs training on mental health. 86.7% of the members said that they knew about some cases of DSH or suicide in their locality. 80.7% members considered suicide by pesticide poisoning as a health problem in the block. An 88% of members said that each of Gram Panchayat and Panchayat Samity has a definite role to play in the reduction of pesticide-related DSH and suicide. Maximum number of members (95%) stressed the public awareness on mental health. A 76.7% of members believed that Panchayat has a role in local system of pesticide sale. 60% members were in favor of imposition of restriction on sale, and 40% suggested that there should be strict restriction on sale to young boys and girls. According to the Samity's estimate, there are about 627 pesticide shops including retailers in the block, of which only 4% are licensed. 80% members said that there were no farmers programs on safe use of pesticide organized by the Panchayat. During the last 1 year, about 492 cases were referred to different Panchayats for amicable settlement of their familial conflict including domestic violence. 

### 3.4. Agenda for Community Intervention

Based on the opinion expressed by the farmers and the Panchayat members, the following action plan was formulated with their endorsement: (a) mental health clinic and community outreach service; (b) IEC development for advocacy and training on “suicide and DSH prevention” for health staff, teachers, students, farmers, panchayat, local health care providers (HCPs), and general public; (c) IEC development on “pesticide safety” for farmers and panchayat members; and (d) training in community psychosocial intervention skills for health staff, multipurpose health workers, Panchayat members, HCPs, and members of NGOs and ICDS workers.

### 3.5. Trainings Conducted

Based on the opinions of farmers and GPMs, the following DSH-suicide prevention training was conducted with the development of appropriate training modules and IEC materials for each target group (Table 1). A series of trainings (total 89) with appropriate IEC materials (Table 2) was conducted in the block, involving the following groups: 5 medical officers, 11 nursing staff, 32 MPHWs, 42 ICDS workers, 98 Panchayat members, 84 HCPs, 58 school teachers, 128 farmers, 46 NGO members, 32 Mahila Samity members, 22 pesticide shop owners, and 19 DSH attempters (special group). In addition, 16 public meetings in 7 GPs were also conducted.

Table 3 shows the feedback responses on some of the IEC materials from the target groups. Positive endorsement on culture fairness rating ranged from 78–98% and usefulness rating 80–100%. 

**Figure 12:**
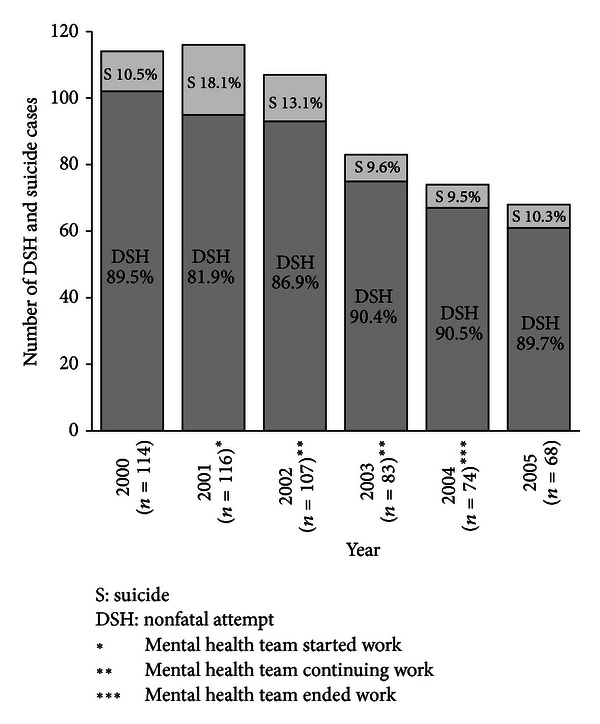
Year-wise distribution (2000–05) of DSH and suicide at Dwariknagar BPHC.

Figure 12 shows the *BPHC admission data:* The analysis of both retrospective (2000-01) and prospective (2002–2005) admission data of DSH (both fatal and nonfatal) at the Dwariknagar BPHC, Namkhana, shows definite declining of incidence of DSH in the block after this preventive activity started.

## 4. Discussion

Participatory research (PR), by definition, involves systematic enquiry in collaboration with those affected by the issue being studied for the purpose of education and taking action or effecting social change [16]. Participatory action research differs from other public health research, because it is based on reflection, data collection, and action that aims to improve health and reduce health inequities through engaging the people who take active participation and actions to improve their own health [17]. Self-harm behaviour is now becoming an urgent public health issue because of its resultant morbidity and mortality and also for its association with both individual and sociocultural factors [18]. For effective prevention, community intervention approaches are promising, as it will promote community participation and enhance responsiveness to this public health priority.

This DSH prevention programme was designed as community-based participatory research (CBPR), because this involvement will help to improve the quality of life and prevention of suicidal behaviours in the community by putting new knowledge in the hands of those who need to make changes. We selected the target groups and devised IEC and training materials as per the community suggestion and endorsement, and they were well accepted by the incumbents. Most of the messages in the posters were the verbatim of local people elicited during the FGDs. Training involving groups from all stockholders in the context of pesticide-related DSH and suicide was quite successful so far in its acceptance and taking responsibility in the preventive process. This approach brought the community members into the research activity as partners, not just subjects. Using the knowledge and opinion of the community to understand concept and ideas about the context and problems relating to suicide and DSH helped us to design activities that guide strategies for objective oriented preventive interventions of suicidal behaviour in the community; one such example is the shared responsibility taking by the MPHW to monitor domestic violence at each household during their scheduled home visits [19]. Active participation of HCPs in referring potential cases to the mental health clinic is another example of their active participation in the preventive process [20]. The findings on licensed pesticide shops are quite alarming, and this issue needs very serious restrictive measures to prevent easy availability of pesticides from the retail shops. It is reported that geographical isolation, economic backwardness, and dearth of health care in rural areas are related with risk factors in suicidal behaviours [21]. These psychosocial issues are quite prevalent in this remote delta region and should be addressed in the DSH-suicide prevention programme.

This is a unique experience of DSH prevention work involving Panchayat and community members in Sundarban. It may be a suitable public health model for DSH prevention in agricultural communities in the developing countries. Success has been reported from community-based suicide prevention programme among some indigenous population [22]. Based on this preliminary success, we think that this model should be enacted in much bigger scale for the whole of Sundarban region, where PR should involve an intersectional approach for preventing suicide and promoting mental health, and that too should actively involve local health system (medical management of poisoning and community poisoning information system and database) and also address government policy (regulation of pesticide distribution at local markets), agricultural support (informing farmers on safe and effective pesticide practices—good pesticide practice), and overall community participation (awareness and public health action through psychosocial intervention) [23].

## Figures and Tables

**Figure 4:**
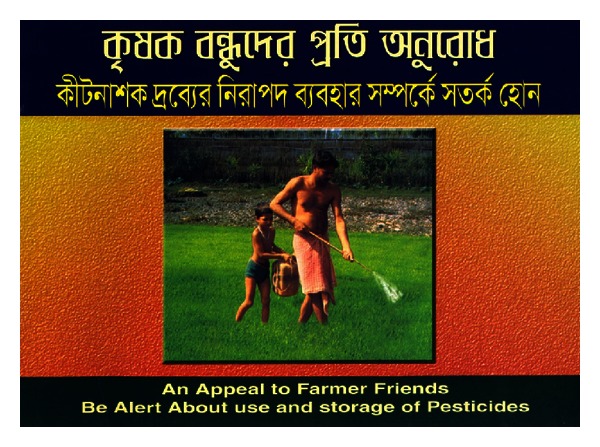
Front page with a picture of local unsafe pesticide practice.

**Figure 5:**
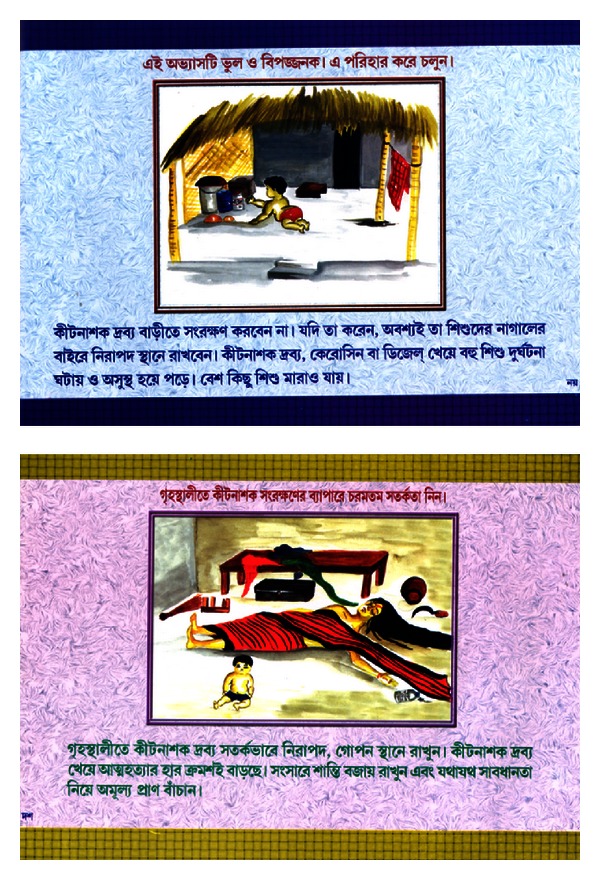
Prevention of accidental poisoning and DSH from unsafe pesticide storage.

**Figure 6:**
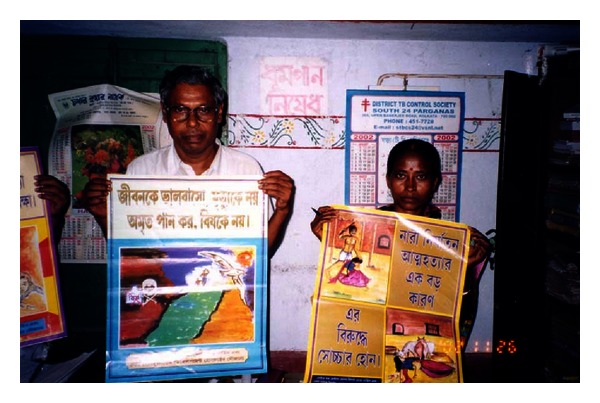
Panchayat members displaying the DSH prevention posters (containing slogans and sketches by local students).

**Figure 7:**
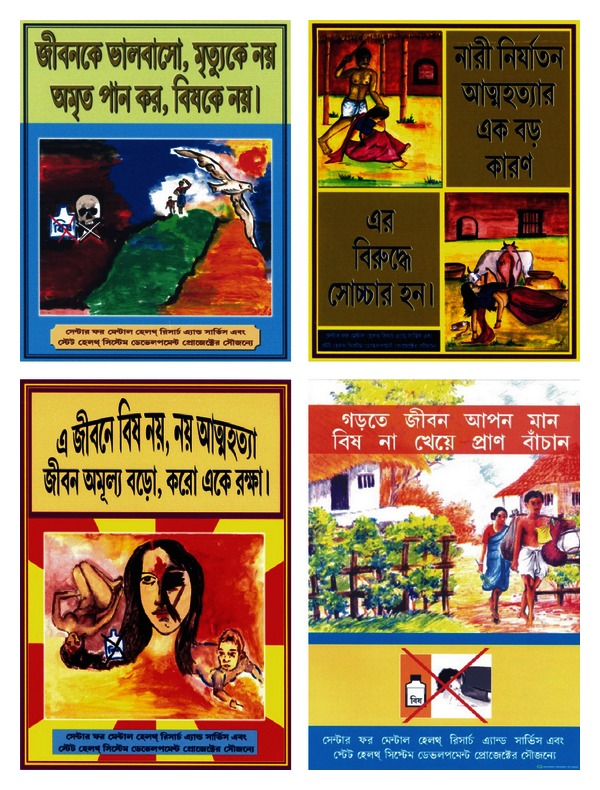
Posters on prevention of DSH suicide and violence towards women.

**Figure 8:**
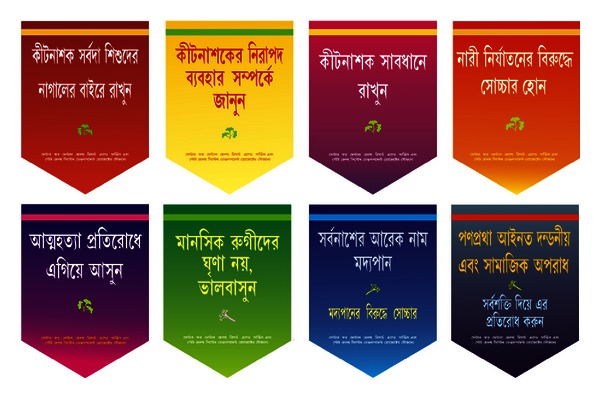
Stickers on DSH-suicide prevention, dowry torture, pesticide safety, and mental health.

**Figure 9:**
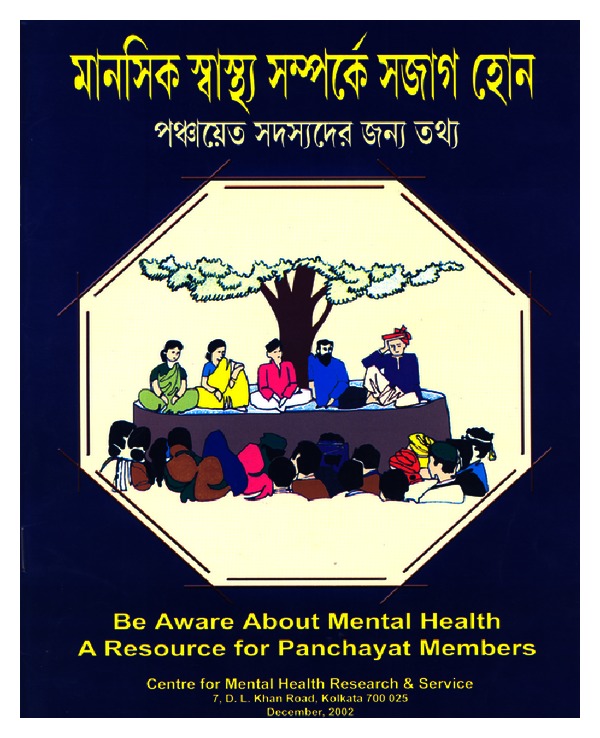
Booklet on “Awareness about Mental Health.”

**Figure 10:**
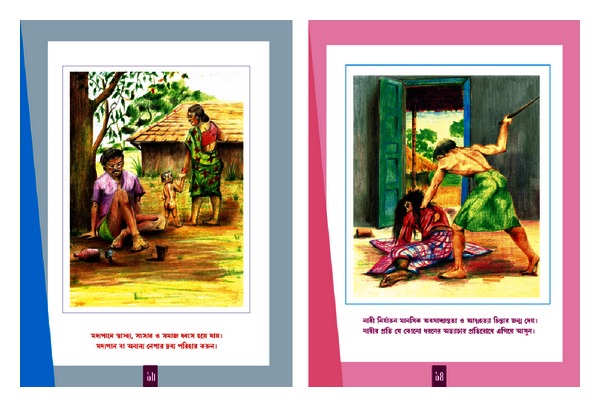
Psychosocial intervention: different local social issues.

**Figure 11:**
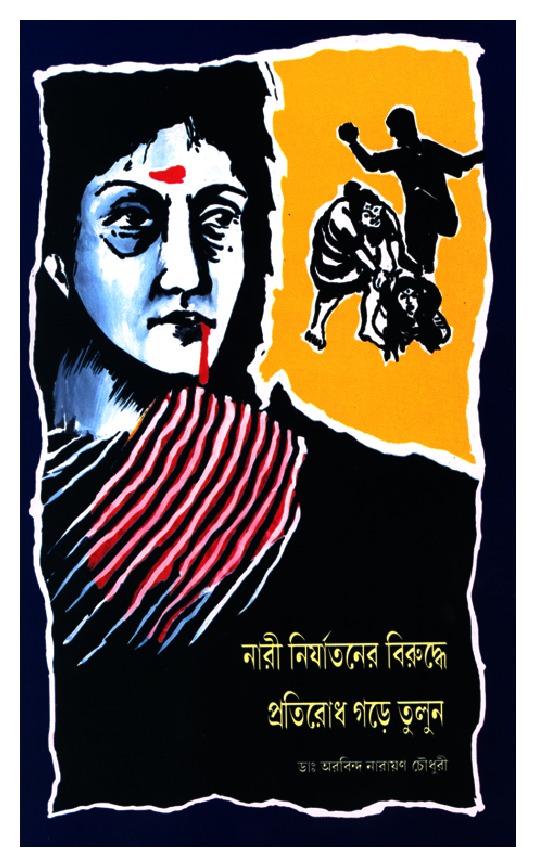
Booklet on prevention of violence towards women (local context).

**Table 1:** Training target groups and associated IEC modules.

Target groups	Specific training module
Medical officers (BPHC and PHC)	Mental health, DSH prevention, and management of poisoning
Nursing staff (BPHC and PHC)	DSH management and followup
Multipurpose health workers and ICDS workers (whole block)	Identification of mental illness and psychosocial intervention, monitoring, and followup home visits
Panchayat members (whole block) Local health care providers (HCPs—whole block) Local school teachers (secondary schools)	Identification of mental illness and psychosocial intervention, common mental disorders, and DSH prevention
Farmers (group from each 7 GPs) Local NGO (9) members Pesticide shop owners in each GP	Pesticide safe storage, correct pesticide use, caution in pesticide sale, and DSH prevention

**Table 2:** Main IEC materials for training.

IEC type	Title	Content	Target groups
Booklet: illustrated 12 paged coloured (Figures 4 and 5)	An appeal to farmers—be alert about the use and storage of pesticides	Examples of unsafe P use in the community/safe P use and storage measures	Farmers/Panchayat

Folder: 4-paged leaflet with guidelines	Safety measures to be used in P use	Hazards of excess P use/precautions during P use/first aid in P poisoning	Farmers/P shop owners/GP members

Coloured posters—8 (Figures 6 and 7)	Pesticide cycle Accidental poisoning DSH/suicide Domestic violence Alcohol dependence	Effects of P on health, food, environment/promote mental health/prevent DSH	Farmers/P shop owners/displayed in All GPs/schools/prominent locations

Stickers: 10 brightly coloured danglers (Figure 8)		Short slogans on safe P use/promotion of mental health/DSH prevention	Display in public places/schools/Panchayat and Mahila Samity office

Booklet—20 pages (Figure 9) and folder on mental health	Awareness about mental health	Mental illnesses and DSH/suicide—how to identify and take action	GP members/BPHC, PHC health staffs/NGOs/HCPs/teachers

WHO booklet—translated into Bengali	5 titles on DSH/suicide	Role of public/GPs/health personnel in suicide prevention	Do

Booklet on DSH prevention—a training material (Figure 10)	Prevent self-harm/psychosocial intervention	DSH and suicide: risk factors/mental illness/local psychosocial stressors/safe pesticide storage/use	Do + farmers/school teachers/Mahila Samity

Booklet—16 pages (Figure 11)	Prevention of violence towards women	Discussed the issue of domestic and other violence to women in local context and how to prevent these	GP members, NGOs, health staff, HCPs, Mahila Samity, teachers

P: pesticide.

**Table 3:** Feedback response of the target groups on each IEC material.

IEC title	Target group	Culture fairness	Useful
An appeal to farmers—be alert about the use and storage of pesticides	Farmers (*n* = 84)	98%	100%
Safety measures to be used in pesticide use	Pesticide shop owner (*n* = 20)	82%	80%
Book and folder on mental health	MPHW (*n* = 30)	92%	100%
Book and folder on mental health	HCPs (*n* = 116)	78%	98%
Book and folder on mental health	Panchayat members (*n* = 80)	84%	95%
Prevent self-harm/psychosocial intervention	Panchayat members (*n* = 87)	98%	100%
WHO booklet—for teachers	School teachers (*n* = 40)	90%	100%
